# Algorithmic opacity in opioid risk scoring and the need for transparent AI regulation

**DOI:** 10.1038/s41746-026-02491-y

**Published:** 2026-02-24

**Authors:** Sherry Yun Wang, Ryan Stofer, Zhouzhou Chu, Xiao Huang, Ang Li

**Affiliations:** 1 School of Pharmacy, Chapman University, Irvine, CA USA; 2 Department of Environmental Sciences, Emory University, Atlanta, GA USA; 3 Department of Electrical and Computer Engineering, University of Maryland, College Park, MD USA

**Keywords:** Computational biology and bioinformatics, Diseases, Drug discovery, Health care, Medical research

## Abstract

NarxCare®, a proprietary opioid risk scoring system embedded in Prescription Drug Monitoring Programs (PDMPs), has generated significant patient complaints. We adhered to the technical specifications and applied them to PDMP and IQVIA PharMetrics® Plus Closed Health Plan claims database. Despite adding socioeconomic covariates, precision (0.01–0.32) was far below the reported benchmark of 0.75, and F1 scores (0.02–0.39) were also substantially lower than the benchmark value of 0.65, across all our reconstructed models.

Machine learning (ML) tools are increasingly embedded in U.S. clinical workflows, yet their opacity raises persistent concerns about fairness, transparency, and regulatory oversight. One such class of tools, opioid risk scoring (ORS) systems, has become central to opioid stewardship strategies across the United States^1^. Among them, NarxCare®, a proprietary algorithm developed by Bamboo Health, is the most widely adopted and currently integrated into statewide Prescription Drug Monitoring Programs (PDMPs) in over 20 states^2^. Despite this broad integration and its growing influence over treatment decisions, NarxCare® remains a black box.

In September 2023, Bamboo Health released technical documentation^3^ revealing that NarxCare®’s ORS calculates risk scores using a basic logistic regression model, with predictive features such as the number of prescribers, the number of dispensing pharmacies, and thresholds of daily Morphine Milligram Equivalents (MMEs) over varying time windows. This raises the broader question of whether commonly used prescription- and claims-based data sources^3^, paired with publicly disclosed documentation for modeling pipelines, are sufficient to support reliable prediction of opioid-related harms across diverse real-world populations.

## Reconstructing NarxCare’s published model

To explore this, we attempted to approximate NarxCare®’s ORS using two independent datasets: the IQVIA PharMetrics® Plus (2006–2022) and California’s PDMP (2010–2023). We engineered features consistent with NarxCare®’s documentation^3^, including cumulative opioid dosage, prescriber count, and pharmacy switching behavior, and trained supervised ML models (logistic regression, random forest, XGBoost, and neural networks). Models were trained to predict either receipt of medication for opioid use disorder (MOUD), as a proxy for opioid use disorder (OUD) in PDMP data^4–6^, or broader opioid-related adverse events defined by International Classification of Diseases (ICD) codes^7^ in the IQVIA dataset (Supplementary Tables 1–3). We used stratified 5-fold cross-validation, applied a range of class balancing techniques^8^ [Synthetic Minority Oversampling Technique (SMOTE), random undersampling (RUS), and edited nearest neighbors (ENN)]. We also incorporated expanded feature sets, including individual-level characteristics (e.g., age, gender, and payment type) and neighborhood-level social determinants of health (SDoH), e.g., median age, income, education, disability, and racial/ethnic composition. This study was deemed exempt from institutional review board approval because it involved unidentifiable data.

## Benchmark performance discrepancies

**Fig. 1:**
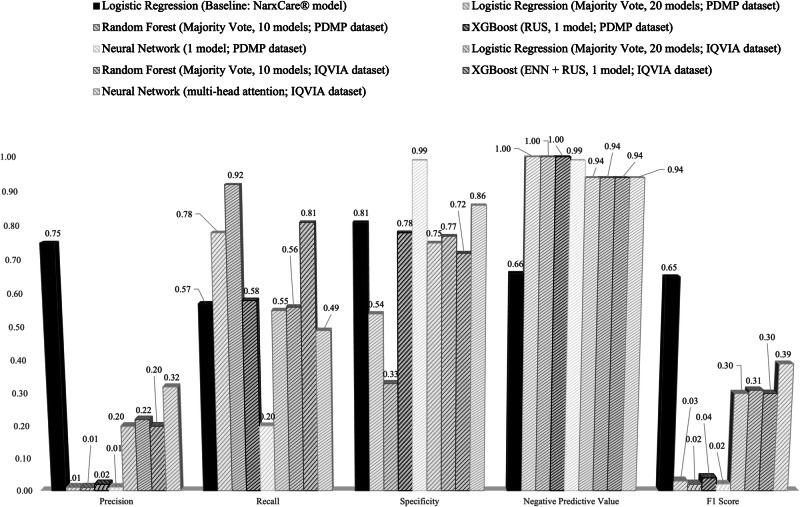
Comparative performance of reconstructed models across PDMP and claims datasets. This figure compares precision, recall, specificity, NPV, and F1 scores across logistic regression, XGBoost, neural networks, and ensemble models trained on PDMP and IQVIA datasets using the NarxCare®-aligned feature set, with class-balancing methods including SMOTE, RUS, and ENN. Each panel presents the best-performing model from each model family, selected across all hyperparameter configurations; detailed results for all models are reported in Supplementary Table 3, enabling direct comparison with the NarxCare® benchmark metrics.

Since NarxCare® trained its model on PDMP data from a Midwestern state^3^, we anticipated comparable, or even improved performance when applying the same approach to our PDMP dataset. However, despite reproducing the published feature set, adding expanded SDoH covariates, and testing multiple modeling strategies (logistic regression, XGBoost, neural networks) with class-balancing techniques (SMOTE, RUS, ENN), all models showed only modest performance across both datasets. For each metric, we report the best-performing value within each model family across all hyperparameter configurations (Fig. 1). The NarxCare® baseline model is shown for reference (Figure 1), with its missing F1 value manually derived from its reported precision and recall. Full results are available in Supplementary Table 3.

Our reconstructed models consistently yielded precision values substantially lower than those reported by NarxCare®, and we acknowledge several important limitations that constrain direct comparability. PDMP-based identification of OUD relies on initiation of MOUD, which does not capture transitions into disease and primarily reflects observed treatment access and prescribing behavior, while claims-based datasets such as IQVIA do not include Medicaid beneficiaries. Algorithms trained on PDMP data may systematically underrepresent patients engaged in opioid treatment programs (OTP)-based care, which has implications for both performance estimates and equity considerations. Taken together, these structural limitations indicate that opioid risk algorithms intended for use across broad patient populations may underperform when trained on incomplete or nonrepresentative data. This discrepancy further suggests that NarxCare®’s reported performance may depend on additional engineered features, undocumented preprocessing steps, or proprietary training optimizations that are not publicly disclosed. Our analysis does not assert causal inference or claim incorrectness of NarxCare®’s outputs or training methods; rather, it highlights the broader risks associated with opaque clinical algorithms that influence high-stakes decisions without independent validation. NarxCare® thus exemplifies a structural transparency challenge: because key elements of the model are proprietary, independent researchers, clinicians, and state agencies cannot determine the sources of observed performance discrepancies or systematically assess generalizability, fairness, or safety.

## Consequences for clinical algorithm oversight

The U.S. Food and Drug Administration (FDA) has historically struggled to delineate its authority over Clinical Decision Support (CDS) tools that do not make explicit treatment recommendations. Tools like NarxCare® fall outside the current definition of Software as a Medical Device (SaMD), allowing them to bypass premarket review. Yet their influence is undeniable: reports of patients being denied pain treatment due to ORS flags are mounting, particularly in marginalized communities^2,9–11^. When the FDA cleared the Apple Watch in 2018 for detecting irregular heart rhythms, many worried the agency would get stuck trying to reverse-engineer the device^12^. Instead, Apple provided extensive firm-based validation data: large-scale clinical studies, clear descriptions of the underlying datasets, transparent reporting of model performance across demographic groups, and evidence that the device performed reliably in real-world conditions. This allowed the FDA to evaluate not only the product but also the company’s development process, documentation practices, and data integrity, consistent with how the agency assesses SaMD^12^. A similar framework would benefit high-stakes CDS systems such as NarxCare®, which generate proprietary risk scores that clinicians may rely upon but may not allow users to independently review the basis for the recommendation, a key criterion for exclusion from device regulation under section 520(o)(1)(E) of the FD&C Act. ^13^ In fact, the FDA explicitly states that software functions that provide a risk probability or risk score for a disease or condition do not meet Criterion 3 and are therefore device functions requiring oversight. This mismatch highlights the regulatory gap: NarxCare® has a device-like influence on clinical care, yet it is not necessary to demonstrate the type of transparent, evidence-based validation or a replicable evidence base that would enable independent assessment of safety, fairness, or generalizability.

While the 21st Century Cures Act of 2016 and the FDA’s 2022 guidance attempted to clarify regulatory boundaries, ambiguity remains, and some developers have reportedly designed their tools to avoid triggering regulatory thresholds^14,15^. Meanwhile, federal policy is shifting. A January 2025 executive order directed agencies to remove barriers to AI innovation, and the White House’s “Winning the AI Race: America’s AI Action Plan^16^” now places healthcare at the center of national AI transformation efforts. Its provisions^16^ include FDA regulatory sandboxes, healthcare AI testbeds, and Centers of Excellence designed to vet AI tools in real-world clinical environments. This moment presents a critical opportunity. A modernized, risk-based regulatory framework is needed to calibrate oversight to clinical impact and align with the SaMD model. Regulatory sandboxes should enable iterative development while ensuring transparency, reproducibility, and safety. Without such mechanisms, tools like NarxCare® may continue to influence clinical decision-making without sufficient transparency unless oversight mechanisms are implemented, raising concerns about potential algorithmic harm in the absence of transparent oversight, even when implemented as part of innovation efforts. Healthcare AI should not be exempt from scrutiny. As national infrastructure evolves to support innovation, we must also build the regulatory and ethical frameworks necessary to protect patients and uphold the integrity of clinical practice.

## Methods

### Dataset

This study leveraged two independent large-scale healthcare datasets to replicate and evaluate the NarxCare® opioid risk scoring algorithm: California’s PDMP and the IQVIA PharMetrics® Plus Closed Health Plan claims database. The California PDMP, accessed via the Controlled Substance Utilization Review and Evaluation System (CURES), comprises de-identified patient-level dispensing records for all Schedule II–V controlled substances, including opioids. It captures prescription-level details such as drug name, strength, quantity dispensed, days’ supply, prescriber and dispensing pharmacy identifiers, fill date, and patient demographics (age, gender, payment type, ZIP5). The IQVIA PharMetrics® Plus Closed Health Plan claims database contains longitudinal adjudicated medical and pharmacy claims from a national sample of commercially insured and Medicare Advantage enrollees, with information on National Drug Codes (NDCs), fill dates, quantities dispensed, prescriber identifiers, ICD-9/10 diagnostic codes, procedure codes, and insurance type. Unlike PDMP, IQVIA does not include pharmacy identifiers. For contextual enrichment, IQVIA ZIP3 codes were mapped to ZIP5 using the SimpleMaps crosswalk^17^, and ZIP5-level socioeconomic indicators (i.e., median age, socioeconomic status, housing, education, employment, disability, and racial/ethnic composition) were appended from the SimpleMaps crosswalk^17^. This enabled integration of neighborhood-level SDoH as a proxy for individual SDoH^18^ into predictive modeling (Supplementary Table 1, Supplementary Table 3, and Fig. 1). Following data cleaning and preprocessing, the CURES dataset yielded approximately17.9 million training observations, 8.9 million validation observations, and 6.7 million testing observations. The IQVIA dataset yielded around 1.03 million training, 0.26 million validation, and 0.32 million testing observations (Supplementary Table 2).

### Feature construction

Feature construction was aligned with Bamboo Health’s published NarxCare Application Overview (2023)^3^, implementing temporal-aggregate measures that mirror the original model’s inputs. Core features^3^ included cumulative MME over the past 365 days and 2 years; total MME dosage in the past 2 years and ≥1 year before the index date; the number of prescriptions with daily MME > 120; and counts of unique prescribers over 2 years and the past 180 days. In the PDMP dataset, an additional behavioral feature, the number of distinct pharmacies dispensing opioids in the prior 2 years, was incorporated. All features were engineered relative to an index date defined as the most recent opioid prescription before the observation window, with sliding-window aggregation implemented using optimized, vectorized routines in Python. ZIP5-linked SDoH variables were appended to capture contextual socioeconomic influences. Continuous features underwent z-score normalization, while categorical variables were one-hot encoded before model training. NarxCare’s baseline model, as disclosed in its technical documentation^3^, was trained via logistic regression on a case-control dataset comprising over 5000 autopsy-adjudicated unintentional overdose deaths matched by age and gender to 500,000 patients prescribed controlled substances who did not die from overdose in a Midwestern state’s PDMP data. Our models were trained to predict opioid-related outcomes available within each data source. In the PDMP dataset, receipt of medication for OUD (MOUD; e.g., buprenorphine, methadone) was used as a pragmatic proxy for OUD, consistent with prior literature^19–21^, recognizing that PDMP data lack ICD-coded diagnostic information and therefore cannot directly capture clinically validated OUD or overdose outcomes. Because methadone administered through federally regulated OTP may not be comprehensively reported to PDMP systems due to 42 CFR Part 2 confidentiality protections, PDMP-observable MOUD primarily reflects pharmacy-dispensed treatments and likely under-ascertains OTP-based methadone treatment. In the IQVIA dataset, opioid-related adverse event labels were assigned based on the presence of ICD-9/10 diagnosis codes^7^ for opioid-related adverse events recorded at any time following the index prescription. Our study cannot determine why NarxCare’s reported performance exceeds what can be reproduced using the publicly stated feature set. Across both datasets, our results show that, even after incorporating additional covariates (e.g., SDoH), conducting extensive hyperparameter optimization, and applying multiple imbalance-mitigation strategies, no reasonable reconstruction of the published feature space achieves performance close to NarxCare’s benchmarks. These discrepancies highlight the possibility that (a) additional, undisclosed features or preprocessing steps could have influenced NarxCare’s reported performance, (b) preprocessing steps or transformations that are not documented, or (c) potential data leakage arising from the internal construction of case-control sets or temporal windows. To ensure a rigorous evaluation, we first trained models using PDMP data, where MOUD initiation served as a proxy for opioid use disorder due to the absence of ICD-based diagnostic information. We then trained models on the IQVIA dataset, which includes ICD codes for opioid-related adverse events, allowing us to evaluate the same feature family under a more clinically specific outcome definition. In both datasets, we implemented multiple strategies to address class imbalance and maximize model performance within the limits of the available information. Despite these efforts, the performance of all reconstructed models remained far below NarxCare’s self-reported metrics. California’s PDMP is not currently integrated with electronic health records (EHR), which limits its capacity to ascertain clinically validated opioid-related adverse events accurately. Given these limitations in outcome ascertainment, we retrained the model using the IQVIA dataset, which contains structured ICD-coded diagnostic data, enabling more robust and clinically grounded labeling of opioid-related adverse events. However, both datasets demonstrated significant class imbalance in the outcome. MOUD initiation can contribute to an underestimation of the actual probability of opioid-related adverse events. In the PDMP dataset, initiation occurred in approximately 1% of patients, indicating a highly imbalanced class distribution. The IQVIA dataset exhibited a more moderate imbalance, with a positive-to-negative case ratio of approximately 1:8. To address this, we employed a range of strategies, including class rebalancing techniques (e.g., oversampling of the minority class, under-sampling of the majority class), as well as deep learning models optimized for imbalanced data. Despite these efforts, model precision did not approach the reported NarxCare® benchmarks, though contextual differences in datasets and outcome definitions limit direct comparability.

### Model training

Following the NarxCare baseline design, a logistic regression model with L2 regularization was implemented as the primary replication model (Supplementary Table 3), providing methodological comparability to the original algorithm. To investigate whether alternative architectures could better capture non-linear interactions, we also trained Random Forests, Extreme Gradient Boosting (XGBoost), feedforward neural networks, wide and deep hybrid architectures, and self–attention–augmented networks (Details shown in Supplementary Table 3). Training and testing sets were generated by randomly splitting the entire dataset to evaluate model performance. Hyperparameter optimization was conducted using grid search with Optuna as the primary tuning strategy, integrated with nested cross-validation within the training partition to ensure reliable and generalizable parameter estimates. We evaluated both hard voting (majority class selection) and soft voting (probability averaging) ensemble strategies, ultimately adopting soft voting due to its superior performance and finer discrimination from aggregating predicted probabilities rather than binary class outputs.

### Evaluation metrics

Model performance was assessed using precision, recall, specificity, negative predictive value (NPV), and F1 score (Supplementary Table 3). All metrics were selected to enable comparison with the baseline model reported by NarxCare®. Both datasets exhibited outcome class imbalance, particularly PDMP, where MOUD initiation occurred in approximately 1% of patients and IQVIA, with a more moderate imbalance for opioid-related adverse events (~1:8). To address this, we implemented multiple imbalance-mitigation strategies, including SMOTE, RUS, and ENN.

## Supplementary information

Supplementary Tables

## Data Availability

The CURES dataset is available upon request from the Department of Justice. The census data can be obtained from the US Zip Codes Database (Pareto SoftwareTM, version 2023). Concerning access to and use of the IQVIA PharMetrics® Plus for Academics dataset, which is licensed to Chapman University under the terms of its agreement with IQVIA Inc.

## Code availability

The code is publicly accessible at https://github.com/Sherry-Yun-Wang/Algorithmic-Opacity-in-Opioid-Risk-Scoring-Need-for-Transparent-AI-Regulation-in-Healthcare.
