# Supplementary material for: Algorithmic opacity in opioid risk scoring and the need for transparent AI regulation
Source: NPJ Digit Med. 2026 Feb 24;9:341. doi: 10.1038/s41746-026-02491-y (PMC13125339; doi:10.1038/s41746-026-02491-y)
Supplement: Supplementary file 1 — Supplementary Tables [file 41746_2026_2491_MOESM1_ESM.pdf]

Supplementary Table 1 Comparison of NarxCare® Baseline Predictive Features with Reconstructed Feature Sets in the CURES and IQVIA Datasets

Feature Name	Category	Description
**Narxcare (baseline)**		Total Morphine Milligram Equivalent (MMEs) prescribed during the most recent 365 days
		Number of pharmacies where narcotics and/or sedatives were filled in the last 2 years
		Total MMEs prescribed during in the last 2 years
		Controlled substance prescriptions with daily MMEs >120 in the last 2 years
		Number of prescribers where narcotics were obtained in the last 2 years
		Total dosage (milligrams) of sedatives prescribed for 1 or more years prior to the current date; Total MMEs prescribed for 1 or more years prior to the current date (including MMEs from MOUDs)
		Number of prescribers where narcotics were obtained in the last 180 days.
**CURES dataset**		
MMEs_under_two_years	Numerical	Total morphine milligram equivalents (MME) prescribed in the last two years.
MMEs_under_one_year	Numerical	Total MME prescribed in the last year.
MMEs_over_one_year	Numerical	Total MME prescribed more than one year ago.
daily_mme_count_above_120	Numerical	Number of days with daily MME exceeding 120.
pharmacies_under_two_years	Numerical	Number of different pharmacies visited in the past two years.
prescribers_under_two_years	Numerical	Number of prescribers visited in the past two years.
prescribers_under_half_year	Numerical	Number of prescribers visited in the past 6 months.
patient_age	Numerical	Age of the patient.
age_median	Numerical	Median age of residents in the patient's ZIP code.
income_household_median	Numerical	Median household income in the patient's ZIP code.
home_ownership	Numerical	Percentage of homeowners in the patient's ZIP code.
education_highschool	Numerical	Percentage of individuals with at least high school education in the patient's ZIP code.
education_college_or_above	Numerical	Percentage of individuals with a college degree or higher in the patient's ZIP code.
unemployment_rate	Numerical	Unemployment rate in the patient's ZIP code.
poverty	Numerical	Percentage of individuals below the poverty line in the patient's ZIP code.
disabled	Numerical	Percentage of disabled individuals in the patient's ZIP code.

race_white	Numerical	Percentage of White individuals in the patient's ZIP code.
race_black	Numerical	Percentage of Black individuals in the patient's ZIP code.
race_asian	Numerical	Percentage of Asian individuals in the patient's ZIP code.
race_native	Numerical	Percentage of Native American individuals in the patient's ZIP code.
race_pacific	Numerical	Percentage of Pacific Islander individuals in the patient's ZIP code.
hispanic	Numerical	Percentage of Hispanic individuals in the patient's ZIP code.
payment_code	Categorical	Type of payment used for prescriptions (e.g., Medicare, Worker's Comp).
patient_gender	Categorical	Gender of the patient (e.g., Male, Female, Other).
**IQVIA dataset**		
MMEs_under_two_years	Numerical	Total morphine milligram equivalents (MME) prescribed in the last two years.
MMEs_under_one_year	Numerical	Total MME prescribed in the last year.
MMEs_over_one_year	Numerical	Total MME prescribed more than one year ago.
daily_mme_count_above_120	Numerical	Number of days with daily MME exceeding 120.
prescribers_under_two_years	Numerical	Number of prescribers visited in the past two years.
prescribers_under_half_year	Numerical	Number of prescribers visited in the past 6 months.
patient_age	Numerical	Age of the patient.
age_median	Numerical	Median age of residents in the patient's ZIP code.
income_household_median	Numerical	Median household income in the patient's ZIP code.
home_ownership	Numerical	Percentage of homeowners in the patient's ZIP code.
education_highschool	Numerical	Percentage of individuals with at least a high school education in the patient's ZIP code.
education_college_or_above	Numerical	Percentage of individuals with a college degree or higher in the patient's ZIP code.
unemployment_rate	Numerical	Unemployment rate in the patient's ZIP code.
poverty	Numerical	Percentage of individuals below the poverty line in the patient's ZIP code.
disabled	Numerical	Percentage of disabled individuals in the patient's ZIP code.
race_white	Numerical	Percentage of White individuals in the patient's ZIP code.
race_black	Numerical	Percentage of Black individuals in the patient's ZIP code.
race_asian	Numerical	Percentage of Asian individuals in the patient's ZIP code.
race_native	Numerical	Percentage of Native American individuals in the patient's ZIP code.
race_pacific	Numerical	Percentage of Pacific Islander individuals in the patient's ZIP code.

hispanic	Numerical	Percentage of Hispanic individuals in the patient's ZIP code.
payment_code	Categorical	Type of payment used for prescriptions (e.g., Medicare, Worker's Comp).
patient_gender	Categorical	Gender of the patient (e.g., Male, Female, Other).

Supplementary Table 2. Descriptive Characteristics of the CURES and IQVIA Datasets for Training, Validation, and Testing Cohorts

Dataset	Variables	Measurements	Training & Validation	Testing
**CURES**	Sample size	Number	Training 17,875,136<br>Validation 8,937,568	6,703,177
	Age	Mean ± SD	MOUD (n = 207,853): 44.71 ± 15.57	MOUD (n = 51,874): 44.70 ± 15.54
			Non-MOUD (n = 26,604,851): 47.05 ± 19.41	Non-MOUD (n = 6,651,303): 47.03 ± 19.41
	Sex	Male	MOUD: 41.89%	MOUD: 41.88%
			Non-MOUD: 43.09%	Non-MOUD: 43.10%
		Female	MOUD: 57.92%	MOUD: 57.90%
			Non-MOUD: 56.81%	Non-MOUD: 56.81%
**IQVIA**	Sample size	Number	Training: 1,034,485<br>Validation: 256,122	322,652
	Age	Mean ± SD	ORAE (n = 133,644): 50.76 ± 18.16<br>Non-ORAE (n = 1,156,963): 41.26 ± 17.01	ORAE (n = 33,411): 50.80 ± 18.11<br>Non-ORAE (n = 289,241): 41.26 ± 17.02
	Sex	Male	ORAE: 46.73%	OUD: 46.92%
			Non-ORAE: 43.95%	Non-ORAE: 44.01%
		Female	ORAE: 53.27%	ORAE: 53.08%
			Non-ORAE: 56.05%	Non-ORAE: 55.99%

Supplementary Table 3. Comparative Performance of Predictive Models for Opioid-Related Outcomes Using prescription drug Monitoring Program (PDMP) and Claims Data

Dataset (features)	Model Type	Precision	Recall	Specificity	NPV	F1 Score

Baseline (NarxCare® predictive features[a])	Logistic Regression (Unknown model)	0.75	0.57	0.81	0.66	0.65
PDMP (NarxCare® predictive features[b])	Logistic Regression (Majority Vote, 10 models)	0.02	0.60	0.71	1.00	0.03
PDMP (NarxCare® predictive features[b])	Logistic Regression (Majority Vote, 20 models)	0.02	0.60	0.72	1.00	0.03
PDMP (NarxCare® predictive features[b])	Logistic Regression (Majority Vote, 100 models)	0.02	0.60	0.72	1.00	0.03
PDMP (NarxCare® predictive features and SDoH[b,c])	Logistic Regression (Majority Vote, 10 models)	0.01	0.78	0.54	1.00	0.03
PDMP (NarxCare® predictive features and SDoH[b,c])	Logistic Regression (Majority Vote, 20 models)	0.01	0.78	0.54	1.00	0.03
PDMP (NarxCare® predictive features and SDoH[b,c])	Logistic Regression (Majority Vote, 100 models)	0.01	0.78	0.54	1.00	0.03
PDMP (NarxCare® predictive features and SDoH[b,c])	Logistic Regression (Majority Vote, 100 models)	0.01	0.78	0.54	1.00	0.03
PDMP (NarxCare® predictive features and SDoH[b,c])	Random Forest (Majority Vote, 10 models)	0.01	0.92	0.33	1.00	0.02
PDMP (NarxCare® predictive features and SDoH[b,c])	Logistic Regression (RUS, 1 model)	0.02	0.63	0.72	1.00	0.03
PDMP (NarxCare® predictive features and SDoH[b,c])	Logistic Regression (ENN + RUS, 1 model)	0.05	0.25	0.96	0.99	0.08
PDMP (NarxCare® predictive features and SDoH[b,c])	XGBoost (RUS, 1 model)	0.02	0.58	0.78	1.00	0.04
PDMP (NarxCare® predictive features and SDoH[b,c])	XGBoost (ENN + SMOTE, 1 model)	0.06	0.09	0.99	0.99	0.07
PDMP (NarxCare® predictive features and SDoH[b,c])	Neural Network (1 model)	0.01	0.20	0.99	0.99	0.02

IQVIA (NarxCare® predictive features[d])	Logistic Regression (Majority Vote, 10 models)	0.20	0.55	0.75	0.94	0.30
IQVIA (NarxCare® predictive features[d])	Logistic Regression (Majority Vote, 20 models)	0.20	0.55	0.75	0.94	0.30
IQVIA (NarxCare® predictive features[d])	Logistic Regression (Majority Vote, 100 models)	0.20	0.55	0.75	0.94	0.30
IQVIA(NarxCare® predictive features and SDoH[d])	Logistic Regression (Majority Vote, 10 models)	0.20	0.55	0.75	0.94	0.30
IQVIA(NarxCare® predictive features and SDoH[d])	Logistic Regression (Majority Vote, 20 models)	0.20	0.55	0.75	0.94	0.30
IQVIA(NarxCare® predictive features and SDoH[d])	Logistic Regression (Majority Vote, 100 models)	0.20	0.55	0.75	0.94	0.30
IQVIA(NarxCare® predictive features and SDoH[d])	Logistic Regression (Majority Vote, 100 models)	0.20	0.55	0.75	0.94	0.30
IQVIA(NarxCare® predictive features and SDoH[d])	Random Forest (Majority Vote, 10 models)	0.22	0.56	0.77	0.94	0.31
IQVIA(NarxCare® predictive features and SDoH[d])	Logistic Regression (RUS, 1 model)	0.20	0.55	0.75	0.94	0.30
IQVIA(NarxCare® predictive features and SDoH[d])	Logistic Regression (ENN + RUS, 1 model)	0.20	0.58	0.73	0.94	0.29
IQVIA(NarxCare® predictive features and SDoH[d])	XGBoost (RUS, 1 model)	0.20	0.60	0.73	0.94	0.30
IQVIA(NarxCare® predictive features and SDoH[d])	XGBoost (ENN + RUS, 1 model)	0.20	0.81	0.72	0.94	0.30
IQVIA(NarxCare® predictive features and SDoH[d])	XGBoost (ENN + SMOTE, 1 model)	0.28	0.17	0.95	0.91	0.21

IQVIA(NarxCare® predictive features and SDoH[d])	Neural Network (1 model, NN w/ 1 fully connected layer, Learning Rate = 0.01 (100 epochs))	0.20	0.54	0.76	0.93	0.30
IQVIA(NarxCare® predictive features and SDoH[d])	Neural Network (1 model, NN w/ 1 fully connected layer, Learning Rate = 0.001 (100 epochs))	0.20	0.56	0.74	0.94	0.29
IQVIA(NarxCare® predictive features and SDoH[d])	Neural Network (1 model, NN w/ 1 fully connected layer, Learning Rate = 0.001 (200 epochs))	0.20	0.56	0.74	0.94	0.29
IQVIA(NarxCare® predictive features and SDoH[d])	Neural Network (1 model, NN w/ 3 fully connected layer)	0.21	0.50	0.78	0.93	0.30
IQVIA(NarxCare® predictive features and SDoH[d])	Neural Network (1 model, Simple feedforward: NN w/ 4 fully connected layers, batch normalization, regularization, and dropout)	0.27	0.55	0.80	0.94	0.36
IQVIA(NarxCare® predictive features and SDoH[d])	Neural Network (custom architecture, Wide & Deep NN: Simple Feedforward NN w/ a parallel and additional flow)	0.26	0.58	0.78	0.94	0.36
IQVIA(NarxCare® predictive features and SDoH[d])	Neural Network (multi-head attention)	0.32	0.49	0.86	0.94	0.39
IQVIA(NarxCare® predictive features and SDoH[d])	Neural Network (focal loss, Utilize a focal loss instead of common binary cross-entripy loss.)	0.26	0.37	0.88	0.92	0.31
IQVIA(NarxCare® predictive features and SDoH[d])	Neural Network (SMOTE, Use SMOTE to generate minority synthetic data.)	0.24	0.47	0.83	0.93	0.32
IQVIA(NarxCare® predictive features and SDoH[d])	Neural Network (ensemble vote, Use three submodels to vote.)	0.29	0.38	0.89	0.93	0.33
IQVIA(NarxCare® predictive features and SDoH[d])	Neural Network (1 model, BiGAN)	0.25	0.41	0.89	0.93	0.31

Abbreviation: SDoH: Social Determinants of Health; RUS: Random Undersampling; ENN: Edited Nearest Neighbors; SMOTE: Synthetic Minority Oversampling Technique;

XGBoost: Extreme Gradient Boosting; IQVIA: Refers to a commercial data source (IQVIA™); F1 Score: Harmonic mean of precision and recallNPV: Negative Predictive Value

[a] NarxCare® predictive features include cumulative morphine milligram equivalents (MME) over the past 365 days and 2 years; total MME dosage in the past 2 years and ≥1 year prior to the index date; the number of prescriptions with daily MME >120; and counts of unique pharmacies and prescribers over 2 years and the past 180 days.

[b] The PDMP-based models use receipt of medication for opioid use disorder (MOUD) as a proxy outcome for identifying individuals with opioid use disorder.

[c] Social determinants of health (SDoH) include individual-level variables (e.g., age, gender, and payment type) and neighborhood-level characteristics such as median age, socioeconomic status, housing, education, employment, disability, and racial/ethnic composition.

[d] The IQVIA-based models use opioid-related adverse events identified through International Classification of Diseases (ICD) codes[4] as the outcome.
